# Unresectable auricular squamous cell carcinoma with locoregional metastasis: use of cemiplimab in an immunosuppressed patient^⋆^

**DOI:** 10.1016/j.abd.2022.12.008

**Published:** 2023-08-29

**Authors:** Tatiana Ferreira França, João Renato Vianna Gontijo, Eduardo Ferraz Veloso Junior, Enaldo Melo de Lima

**Affiliations:** a Dermatology Unity, Hospital das Cínicas, Universidade Federal de Minas Gerais, Belo Horizonte, MG, Brazil; b Hospital Mater Dei, Belo Horizonte, MG, Brazil

*Dear Editor,*

An 81-year-old male agricultural worker, with hypertension complicated with nephropathy and renal transplantation in 2005, using sirolimus, was submitted to radiotherapy and hormone blockade therapy in 2017 for prostate cancer. He had a past history of multiple basal cell carcinomas and squamous cell carcinomas (SCCs). He developed a moderately differentiated, ulcerated, and infiltrating SCC in the right auricular pinna (Fig. 1A), considered unresectable due to recurrence after four surgical interventions, with involvement of the perichondrium, cervical lymph nodes, and salivary glands, demonstrated by anatomopathological examination. Screening for distant metastasis through PET-CT was negative, and he was classified as T3N2bM1. He received intravenous cemiplimab, 350 mg every 21 days, and 20 sessions of radiotherapy (RT), 20 fractions of 250 cGy, a total dose of 50 Gy, in the tumor and auricular pinna, with complete involution of the neoplasm and regional involvement in four months (Fig. 1B), evaluated clinically and through a second PET-CT. There were no adverse effects to the medication. He evolved with progression of the prostate cancer, confirmed by anatomopathological examination, and died 14 months after the use of cemiplimab.

**Figure 1:**
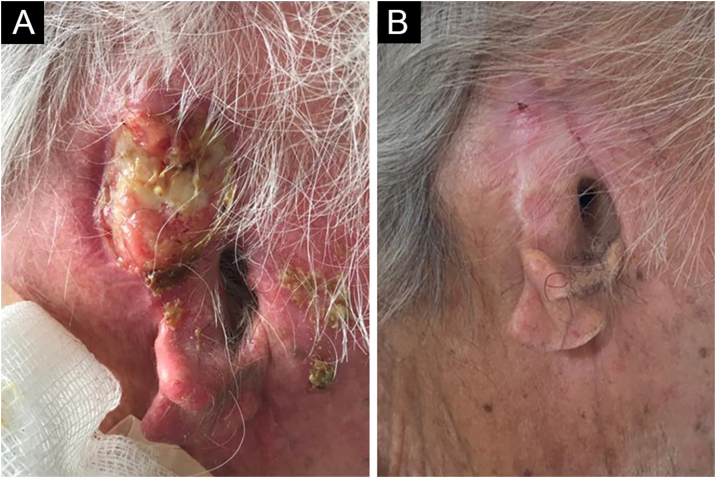
(A) Extensive tumor affecting the auricular pinna and adjacent structures. (B) Cicatricial lesion on the pinna after four months using cemiplimab.

Around 5% of SCCs are classified as advanced neoplasia when they present as locally advanced or metastatic and not amenable to curative surgery and/or curative radiotherapy.^1^, ^2^ Until recently, chemotherapy and epidermal growth factor receptor inhibitors were the only options available for these cases, with low efficacy, nonsustained response rates, and various side effects, being considered palliative treatments.^1^

Cemiplimab is the first approved systemic treatment for SCC that improves survival. This is a fully human anti-PD-1 IgG4 antibody.^3^ An indirect comparison of treatments concluded that it is the systemic therapy with the strongest evidence of clinical benefit for advanced cases and improved survival.^4^

The high mutation burden present in SCCs from exposure to ultraviolet radiation has been associated with the effectiveness of PD-1 inhibition in several advanced solid neoplasms.^1^, ^2^ Furthermore, the strong association between immunosuppression and tumor risk has suggested that immunosurveillance plays an important role in tumor control, and approaches to enhance the anti-tumor immune system may be effective.^1^

PD-1 is a transmembrane inhibitory protein present in immune cells, and blocking it increases the anti-tumor activity of T-cells, promoting immunological control and the death of cancer cells.^3^

It is not possible to precisely determine the isolated effect of the PD-1 inhibitor in the case described herein, due to the concomitant use of RT. However, there was resolution of enlarged cervical lymph nodes that were not irradiated, possibly due to the medication effect. In phase 2 studies with patients with locally advanced SCC, 55% of them also underwent radiotherapy prior to starting the medication.^1^

The advent of new drugs for the treatment of cutaneous neoplasms has shown promising results, with evidence of clinically significant effects, acceptable safety, and tolerability profile in patients with advanced SCC who are not candidates for surgery or RT.^5^ Long-term studies including immunosuppressed patients are required to assess the outcomes in these patients and to determine the efficacy and side effects of cemiplimab in this population.

## Financial support

None declared.

## Authors' contributions

Tatiana Ferreira França: Drafting and editing of the manuscript; critical review of important intellectual content and approval of the final version of the manuscript.

João Renato Vianna Gontijo: Intellectual participation in the propaedeutic and/or therapeutic conduct of the studied cases; drafting and editing of the manuscript; critical review of important intellectual content and approval of the final version of the manuscript.

Eduardo Ferraz Veloso Junior: Intellectual participation in the propaedeutic and/or therapeutic conduct of the studied cases; drafting and editing of the manuscript; critical review of important intellectual content and approval of the final version of the manuscript.

Enaldo Melo de Lima: Intellectual participation in the propaedeutic and/or therapeutic conduct of the studied cases; drafting and editing of the manuscript; critical review of important intellectual content and approval of the final version of the manuscript.

## Conflicts of interest

None declared.
